# The *Myotis chiloensis* Guano Virome: Viral Nucleic Acid Enrichments for High-Resolution Virome Elucidation and Full Alphacoronavirus Genome Assembly

**DOI:** 10.3390/v14020202

**Published:** 2022-01-20

**Authors:** Sebastian Aguilar Pierlé, Gabriel Zamora, Gonzalo Ossa, Aldo Gaggero, Gonzalo P. Barriga

**Affiliations:** 1 Inorevia, Pepinière Paris Santé Cochin, 75014 Paris, France; sebastian.aguilar@inorevia.com; 2 Laboratory of Emerging Viruses, Virology Program, Institute of Biomedical Sciences, Faculty of Medicine, Universidad de Chile, Independencia 1027, Santiago 8380000, Chile; gabriel.zamora@ug.uchile.cl; 3 ConserBat EIRL, Macal Alto s/n, San Fabián, Ñuble 3780000, Chile; chalofoh@gmail.com; 4 Laboratory of Environmental Virology, Virology Program, Institute of Biomedical Sciences, Faculty of Medicine, Universidad de Chile, Independencia 1027, Santiago 8380000, Chile

**Keywords:** surveillance, emerging viruses, coronavirus, virome and bats, bat, virome, zoonosis, alphacoronavirus

## Abstract

Bats are widespread mammals of the order Chiroptera. They are key for ecosystem functioning, participating in crucial processes. Their unique ability amongst mammals to fly long distances, their frequently large population sizes, and their longevity favor infectious agent persistence and spread. This includes a large variety of viruses, encompassing many important zoonotic ones that cause severe diseases in humans and domestic animals. Despite this, the understanding of the viral ecological diversity residing in bat populations remains unclear, which complicates the determination of the origins of zoonotic viruses. To gain knowledge on the viral community of a widely distributed insectivorous bat species, we characterized the guano virome of a native Chilean bat species (*Myotis chiloensis* (Waterhouse, 1840)). By applying a novel enrichment strategy, we were able to secure a consequent percentage of viral reads, providing unprecedented resolution for a bat virome. This in turn enabled us to identify and assemble a new bat alphacoronavirus from Chilean bats closely related to PEDV, an important viral pathogen with high mortality rates in suckling piglets. This study highlights the importance of applying and improving high-resolution virome studies in this vital order to ultimately enhance epidemiological surveillance for potentially zoonotic pathogens.

## 1. Introduction

Bats are mammals in the order Chiroptera that can be found in all continents except the polar regions and some oceanic islands [1]. They are the second most widely distributed mammalian group after rodents and are represented by at least 1400 extant species worldwide [1,2,3]. They are a key group for ecosystem functioning, participating in crucial processes, including pollination and seed dispersal; control through predation of vertebrate and invertebrate populations (comprising pest species), indirectly controlling the composition and structure of plant communities through its effect on herbivory; and recycling of energy, being able to mobilize large volumes of nutrients [4,5]. Additionally, bats are hosts of a wide diversity of parasites and pathogens, and their diversity can have profound implications for the transmission of diseases in ecosystems.

Infectious agents exist in commensal, mutual, or parasitic relationships with their hosts. The relationship is restricted to a limited number of host species due to genetic adaptations developed during co-evolution. Spillage of pathogens between different animal species can occur sporadically. This can result from accidental or deliberate intrusion by one animal species into the ecological niche of another [6].

Bats are unique among mammals in their ability to fly and inhabit diverse ecological niches. Their ability to fly long distances paired with their longevity favor infectious agent persistence and spread [7]. In addition to this, changing environments highlight the shared health risks by humans and bats. These characteristics together with their frequently large populations emphasize their potential as hosts for pathogens [3]. Their role in disease epidemiology is further supported by the diversity of microorganisms detected in their microbiomes such as bacteria, fungi, parasites, and viruses. So far, viruses belonging to at least 14 families have been identified in bats [8]. Previous and ongoing research is predominantly focused on viral agents. This is mostly due to the potential for cross-species contamination, as illustrated by the Ebola outbreak in West Africa from the mid 2000′s and the COVID-19 pandemic [9]. Identification in bats of additional relevant viral pathogens with cross-species transmission potential such as rabies virus [3,10], henipaviruses (Nipah and Hendra viruses), and coronaviruses (CoVs) has further highlighted the importance of monitoring and characterizing the viromes of these key players in a given ecosystem [11,12,13].

CoVs are classified in four different genera: Alphacoronavirus, Betacoronavirus, Gammacoronavirus, and Deltacoronavirus [14]. These viruses can infect humans, domestic and wild mammals and birds, causing respiratory and enteric diseases. Surveillance programs around the world have made an effort to better understand bats’ role as reservoirs for several viruses that pose significant threats to human health, the poultry industry, and hog farming, amongst others. Several different bat species share an ecosystem with humans, for example, *Myotis chiloensis* [15], *Tadarida brasiliensis* [16], and farm animal species, which can favor zoonotic infections [17]. Swine Acute Diarrhea Syndrome Coronavirus (SADS-CoV) and porcine epidemic diarrhea virus (PEDV) are examples of coronaviruses that jumped into piglets, causing acute gastroenteritis in neonatal piglets and ultimately, important economic losses [18]. In silico analyses suggest that SADS-CoV and PEDV may have originated from bat CoVs, reaffirming interspecies transmission and further suggesting the importance of viral surveillance programs in bats [11].

*Myotis chiloensis* is a small Vespertilionid bat species endemic to the southern cone of South America. *M. chiloensis* feeds on insects—primarily nematocerans—that are captured in flight [19,20]. They are currently considered a “Least Concern” species by the International Union for Conservation of Nature [19]. *M. chiloensis* is usually found cohabitating with other bat species, in particular with the Brazilian free-tailed bat (*Tadarida brasiliensis*), the big-eared brown bat *(Histiotus macrotus*), the small big-eared brown bat (*H. montanus*), and the southern big-eared brown bat (*H. magellanicus*) [20,21,22]. Although some studies have described the bacterial and fungal [2,23] communities that they harbor, little is known about the virome of these widely distributed insectivorous bats.

**Figure 1:**
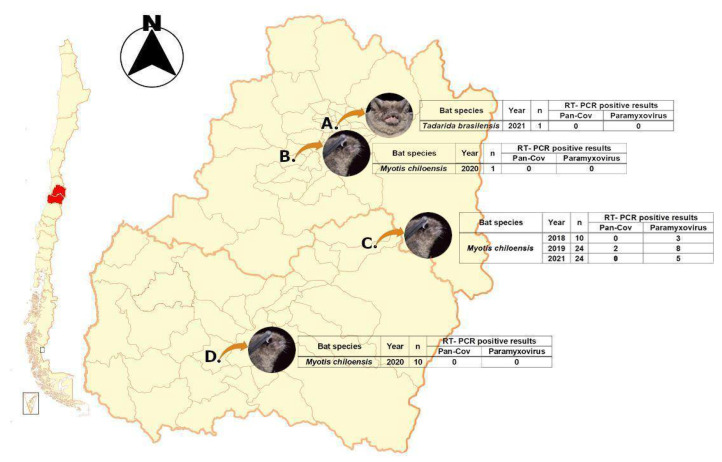
Sampling locations. Letters corresponding to the specific locations were collected. Location C was monitored from 2018–2021 with positive results to CoV and PMX.

In order to gain knowledge on the viral community in a widely distributed insectivorous bat species, we characterized the guano virome of *M. chiloensis*. We monitored a bat colony located in the O’Higgins region of Chile from 2018–2021 (Figure 1). By applying a combined enrichment strategy, we were able to secure a consequent percentage of viral reads. This entailed pairing flocculation for the enrichment of viral particle concentration, molecular enrichment through automated depletion, and bioinformatic filtering of contaminant sequences. Emerging infectious diseases of zoonotic origin embody a major public health and animal industry challenge. Here we describe a new bat alphacoronavirus from a Chilean bat closely related to PEDV an important viral pathogen with high mortality rates in suckling piglets, whose zoonotic potential should be further analyzed. We also highlight the importance of maintaining viral surveillance in this field.

## 2. Material and Methods

Sample collection: Fecal samples were obtained from bat colonies established in human buildings between the Metropolitan Region (MR) and O’Higgins Region (VI) of Chile at different dates between 2019 and 2021 (Figure 1). They were stored at −80 °C. It should be noted that both samples from location C collected in 2019 were pooled for sequencing in order to get an overview of the colony’s virome.

PCR screening: Fecal samples were homogenized in viral transport medium (VTM) and centrifuged at 6000× *g* for 15 min at 4 °C, and supernatant was stored at −80 °C. Viral RNA was extracted from 250 µL of the supernatant obtained with TRIzol^®^ Reagent (Thermo Fisher Scientific Waltham, MA, United States) based on the method of Chomczynski and Sacchi [24]. The RNA obtained was stored at −20 °C. Coronavirus screening was performed by a pancoronavirus one-step RT-PCR based on primers for a 251 bp conserved region in the polymerase gene (Cor-FW 5′-ACWCARHTVAAYYTNAARTAYGC−3′; Cor-RV 5′-TCRCAYTTDGGRTARTCCCA−3′) [25]. RT-PCR was made with RT-PCR KAPA SYBR^®^ FAST one-step kit (KAPA Biosystems, Wilmington, MA, United States). [25]. The PCR product was run on a 1.5% agarose gel in TAE 1x at a constant 80 V for 30 min. Paramyxovirus screening was made by a panparamyxovirus one-step RT-PCR based on primers for a 121 bp conserved region in domain III of the RNA-dependent RNA polymerase gene (PMX1-FW 5′-GARGGIYIITGYCARAARNTNTGGAC−3′; PMX2-RV 5′-TIAYIGCWATIRIYTGRTTRTCNCC−3′) [26]. RT-PCR was performed with Brilliant III Ultra-Fast qRT-PCR Master Mix (Agilent Technologies, Santa Clara, CA, United States). PCR product was run on a 2.5% agarose gel in TAE1x at a constant 80 V for 30 min.

Viral enrichment: We weighed 1 g of bat guano and resuspended it until reaching 5 mL of PBS (the ratio was 1:5, buffer was previously autoclaved and filtered using 0.2 µM esterase cellulose filters). Samples were vortexed vigorously, then centrifuged at 2000× *g* for 2 min at 4 °C, taking the supernatant. Next, the supernatant was centrifuged at 400× *g* for 10 min at 4 °C; taking once again the supernatant, this step was repeated once [27]. Next, the sample was centrifuged at 16,000× *g* for 45 min and the supernatant was kept. Samples were then filtered using a 0.2 µM sterivex made out of PES using a 50-milliliter syringe. Samples were then treated with DNase (Goldenbio, Montclair, CA, United States, 100 U/mL of the original sample (from step 1), 100 U/mL RNAse (Goldenbio) A, and 100 U/mL Turbonuclease (Sigma, San Luis, MO, United States). Next, samples were incubated at 37 °C for 1.5 h or at 4 °C O/N. RNA shield was then added for enzyme inactivation. Samples were concentrated using PEG-NaCl and spinning at 4000× *g* for 30 min and then through ultracentrifugation at 100,000× *g* for 1 h. Total RNA was then extracted using the QIamp viral RNA extraction kit. Total RNA QC check and quantification using the nanochips for the bioanalyzer and finally, concentration was measured using Invitrogen’s Qubit.

Depletion and library preparation and sequencing: After total RNA enrichment for viral sequences through centrifugation, total RNA was depleted for rRNA using Illumina’s Ribozero Plus kit with a custom protocol in the Magelia (Inorevia, Paris, France). After bioinformatic verification of probe compatibility for bacterial/mammalian selection, 2 µLs of total RNA were used in the instrument for fully automated molecular enrichment of viral sequences. After depletion, total RNA was run in the Bioanalyzer 2100 using a nano RNA chip to determine rRNA contamination. Next, the total stranded RNA illumina protocol for library preparation was performed using 100 ng of depleted RNA. Library preparation yielded 98.2 nM of high-quality sequenceable material that was then run in a NextSeq 500 Mid Output Kit v2 (150 cycles) cartridge. This yielded a total of 90,530,942 raw reads. The data for this RNA-seq study has been submitted in the GenBank Sequence Read Archive (SRA) under project number PRJNA781397, SUB10683119.

Bioinformatic analyses: Reads were trimmed and read quality was assessed using the QC for sequencing reads tool from the Genomics Workbench (CLC, Qiagen, Paris, France). Mapping and de novo assembly were also performed using this software. Reads were then mapped to three different *Myotis* spp. genome assemblies in order to remove sequences of bat origin. Then, de novo assembly was performed on high quality reads under the following parameters: using updated contigs with an automatic bubble size, minimum contig length = 200, applying automatic word size and scaffolding, the paired distances were automatically detected, the mismatch cost was 2, insertion cost 3, deletion cost 3, length fraction 0.5, and similarity fraction 0.5. Finally, reads were mapped back to the contigs to consolidate them.

The assembled contigs were then automatically blasted under the following parameters using the blast nr database at NCBI: Match/Mismatch and Gap Costs = (Match 2 Mismatch 3 Existence 5 Extension 2), Expectation value = 0.05, Word size = 11, masking low complexity regions, a maximum number of hits at 100 and opening the search to all organisms. Assembly of the viral sequences was additionally manually curated after determining ORFs.

In parallel, sequences were treated using the Kaiju web server for taxonomic classification under the following parameters: we used the NCBInr protein database, low complexity protein sequences were filtered, Geedy and MEM run modes were both applied, minimum match length was 11, and minimum match score was 75 with 5 mismatches allowed.

Phylogenetic analysis: To perform phylogenetic analyses, the full-length sequence of the newly assembled *M. chiloensis* alphacoronavirus 1 was aligned with assorted related viral sequences isolated in different species including bat, murine, feline, porcine, avian, civet, and human origin. A total of 43 sequences were used. The accession numbers can be found in Table S1. The algorithm used for the alignment is the QIAGEN Aarhus applying the “very accurate” setting. The alignment was performed under the following parameters: Gap open cost = 10, Gap extension cost = 1,0, End gap cost = As any other. This alignment was then used in the IQ-TREE webserver to generate a phylogenetic tree. Modelfinder was paired with tree reconstruction and ultrafast bootstrap (1000 and 10,000 replicates). The Best-fit model according to BIC was GTR + F + I + G4. The model of rate heterogeneity was Invar + Gamma with 4 categories. Maximum likelihood and consensus trees were generated. Full results for this analysis can be found in Supplementary file 1, including bootstrap values.

## 3. Results

### 3.1. Collected Samples

Samples were collected from four different locations from two different regions in central Chile. Locations A, B, and D were negative for viral screening; however, location C was positive for paramyxovirus for samples collected in 2018 and 2021. In addition to this, two samples from 2019 were positive for CoV, and these samples were pooled, purified, and ultraconcentrated for molecular enrichment through depletion and NGS library prep/sequencing (Figure 1).

### 3.2. Sample Enrichment and Virome Elucidation/Alphacoronavirus Genome Assembly and Phylogeny

**Figure 2:**
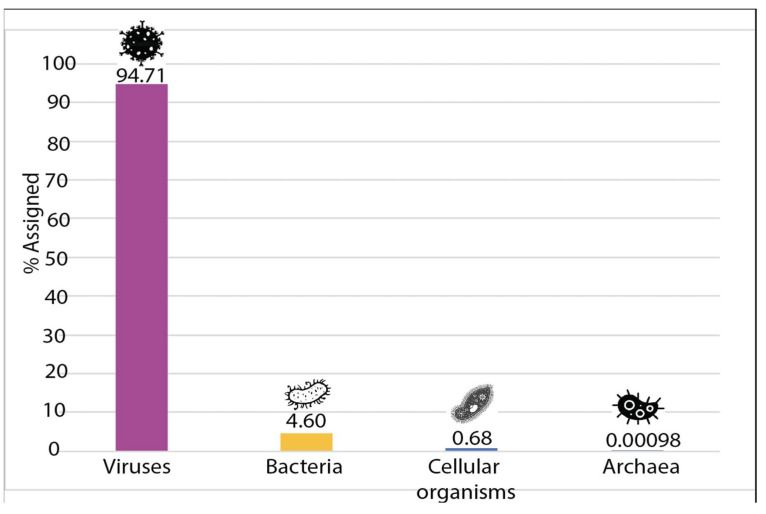
Proportion of the different domains of life represented in this study.

**Figure 3:**
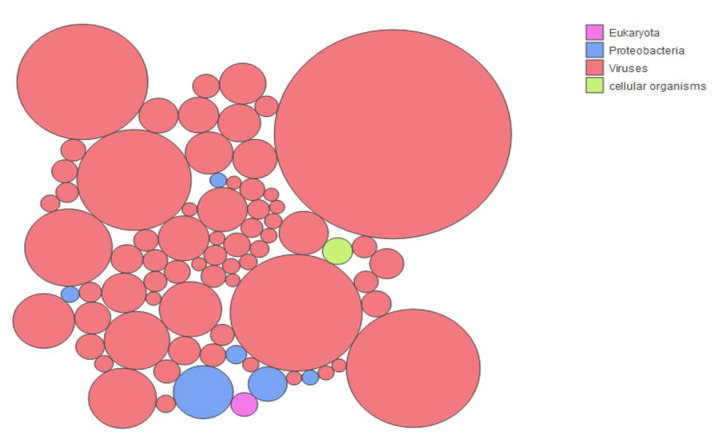
Origin of the taxa with more than 0.1% representation in the data set. Each circle represents one of the taxa that represents >0.1% of the reads in the population. The color attributed to each circle identifies the domain of life to which the taxa belongs, as shown in the legend: pink for eukaryotes, blue for proteobacteria, terracotta for viruses, and light green for cellular organisms (fungi). The figure was generated using the Kaiju web server tool.

Depletion of previously enriched total RNA extracted from guano samples allowed us to reach a 0% rRNA contamination level after Bioanalyzer verification using a Nanochip. After successful library prep and sequencing, a total of 90,530,942 high-quality reads were obtained. These reads were filtered through three different *Myotis* spp. assemblies prior to de novo assembly of these reads. This enabled us to generate a total of 5460 contigs. These contigs were blasted in NCBI’s nr database, significant identity values can be found in Table S2. In parallel, the Kaiju tool was used to identify the different taxa present in the sample, which revealed a total of 2666 different taxa. Surprisingly, the vast majority of the identified taxa were of viral origin. As shown in Figure 2 and Figure 3, 94.71% of the taxa significantly represented in this study were viral, highlighting the success of the combined enrichment procedure used for this study. A total of 4.6% of the taxa were found to be of bacterial origin and less than a total of 1% corresponded to cellular organisms (mostly fungi) and archaea. The full complement of taxa can be found in Table S3. The literature was scanned for studies that attempted to look at bat guano viromes applying a variety of viral enrichment methods [10,28,29,30,31].

Determination of the proportion of total viral sequences allowed us to determine that our strategy enabled us to secure the highest number of viral reads for bat guano virome study to date, to our knowledge (Table 1). In an effort to try to define the representation of the different taxa present in the sample, we looked at the origin of the taxa that represented more than 0.1% of the total sample.

Figure 3 shows that the vast majority (66/74) of the most represented taxa were of viral origin, further supporting the success of the enrichment strategy used for this study. The ten most represented taxa corresponded to insect viruses. This is not surprising considering *M. chiloensis*’ insectivorous diet. These taxa corresponded to Hubei diptera virus 21, Hubei *Tetragnatha maxillosa* virus 8, Hubei mosquito virus 4, Hubei picorna-like virus 61, *Dendrolimus punctatus* tetravirus, *Spodoptera exigua* iflavirus 2, Hubei partiti-like virus 1, Wuhan Millipede virus 4, *Helicoverpa armigera* iflavirus, and Wenzhou tombus-like virus 11. The six most represented bacterial taxa included: *Pseudomonas* spp., *Acinetobacter baumanii*, *Pseudomonas syringae*, *Burkholderia* spp., and two unidentified Gammaprotobacteria. In the two underrepresented categories, one taxon corresponded to a fungal species and another one to a eukaryotic parasite (*Plasmodium ovale wallikeri*) that was previously identified in African bats.

**Figure 4:**
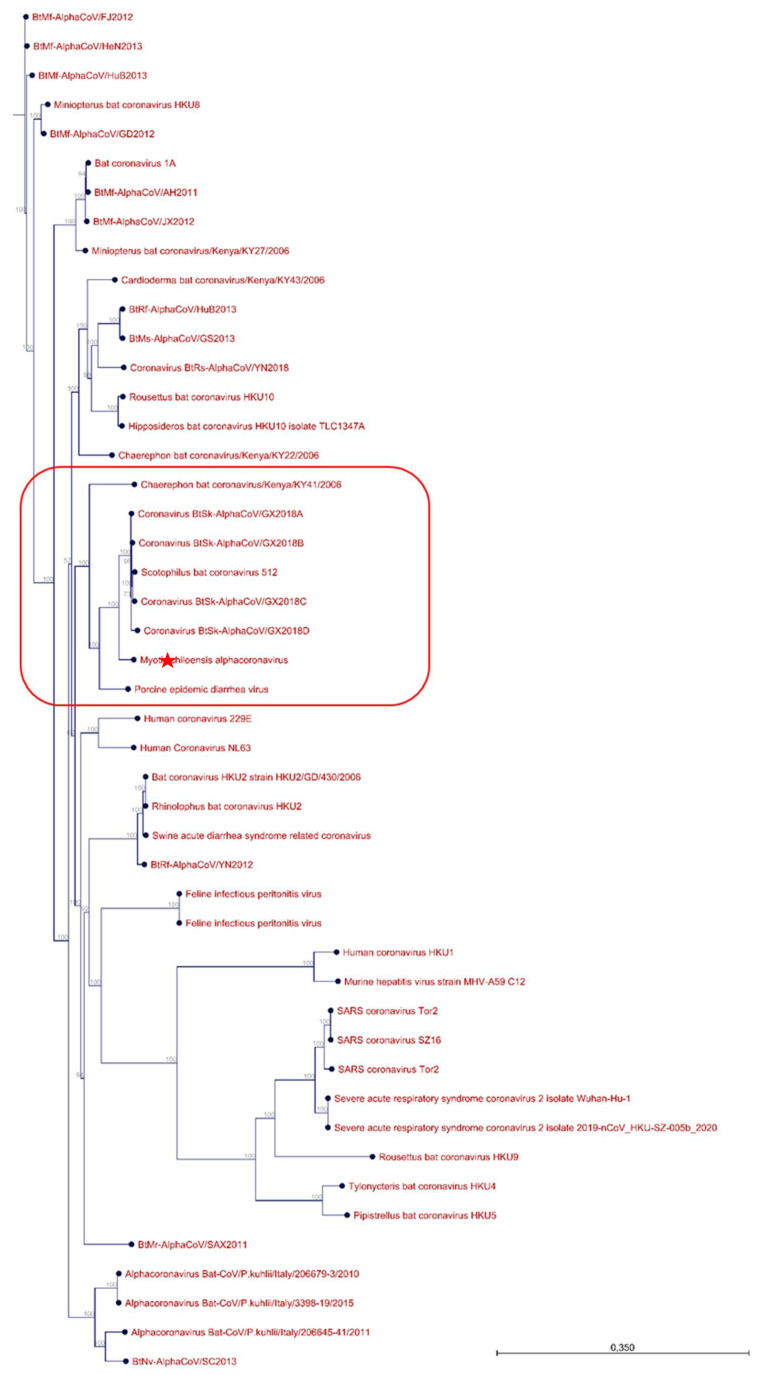
Phylogenetic tree generated including the M. chiloensis viral sequence. The novel *M. chiloensis* viral genome sequence is shown with “red star”, the clade is highlighted in red.

Considering the characteristics of the samples that were processed, including a pancoronavirus and panparamyxo virus PCR positive test and the proximity to humans, we interrogated whether any viruses in this family could be identified among the different taxa. Surprisingly, it was not possible to identify contings or taxa related to paramyxoviruses. However, both strategies enabled us to identify contigs with identities to alphacoronaviruses. On the one hand, the de novo assembly strategy allowed for identification of three contigs with high coverage that were found to have significant identity to bat coronaviruses. These contigs were used for mapping and reassembly of a full genome sequence that was then used for phylogenetic analyses (Figure 4). On the other hand, taxa classification using the Kaiju tool also identified a minimum of 12 contigs with significant identities to coronaviruses (Table S3). Out of these nine showed identities to alphacoronaviruses and five were likely to be of bat origin. These contigs were constructed with a minimum of 200 reads.

The three contigs generated by the de novo strategy were then used for mapping and reassembly in order to see if we would be capable of generating a full genome sequence. The full sequence was 28,323 nucleotides in length, suggesting the full genome sequence was generated. Phylogeny tree was built using 56 related sequences and the newly identified genome (Table S1). As seen in Figure 4, the newly identified genome sequence is grouped with six different bat alphacoronaviruses and a more distantly related Porcine epidemic diarrhea virus, further confirming the presence of this pathogen in this sample. Interestingly, most of the alphacoronaviruses of bat origin that were found in the same clade as the one identified in this study originated from China and one from Kenya and were isolated in insectivorous bats.

## 4. Discussion

Recently, several major outbreaks of emerging viral diseases, including the Hendra, Nipah, Marburg, and Ebola viruses, severe acute respiratory syndrome (SARS), and Middle East respiratory syndrome (MERS), as well as the current COVID-19 coronavirus pandemic, have been reported with increasing frequency [32,33]. Importantly, all these outbreaks have been linked to a suspected zoonotic origin from bat-borne viruses (or at least with bats playing a role as an intermediate host) [11]. Bats, the only mammal capable of powered flight display features that are unique among this animal class, a long lifespan relative to body size, a low rate of tumorigenesis, and an exceptional ability to carry diverse viruses without developing signs of clinical disease. Even though human-to-human transmission of SARS-CoV and MERS-CoV seems to be the most common type of event, some human cases recorded were occasionally the results of zoonotic transmission. This raised interest in the identification of animal reservoirs. In addition, an improved understanding of the natural history of these epidemics, including tracing viral evolution across different hosts, will allow us to improve how we tackle such public health challenges. Evidence suggests that almost all human coronaviruses have zoonotic origins or otherwise have a close relative that circulates in wild animals (bats) and domestic animals (camels and cattle). Besides SARS-CoVs and MERS-CoVs, numerous other CoVs have been detected in bats in Africa, Asia, Europe, and America and are classified into the genera alphacoronavirus and betacoronavirus. The metagenomic view on viruses (virome) unlocks the simultaneous detection of viruses in individual or pooled samples within a very short time. However, for samples with a high host material background, it is becoming apparent that purification procedures are necessary for NGS approaches to significantly increase the number of obtained viral sequences from biological samples.

In our study, we describe for the first time the virome of a previously metagenomically uncharacterized native bat species from Latin America: *M. chiloensis*. Importantly, the characterized colony lived in close proximity to humans, i.e., a church in the O’Higgins region. A combined approach for viral enrichment through benchtop centrifugations and efficient RNA depletion in an automated platform, the Magelia, enabled us to provide insights into the *M. chiloensis* virome with unprecedented resolution. To our knowledge, our strategy provided for the highest proportion of viral sequences in host “contaminated” samples Table 1.

Several strategies have been applied historically to enrich the viral content of guano samples used for virome studies. Centrifugation and filtration are the most commonly utilized strategies. Most studies incorporate these strategies to remove host cells or other micro-organisms. A nuclease-treatment step, where DNAse or RNAse will destroy exogenous nucleic acids but is not thought to affect nucleic acid protected by the viral capsid or envelope can be applied in addition. Finally, ultracentrifugation is also featured as a common method for the concentration of viruses from samples, applied in addition. Finally, ultracentrifugation is also featured as a common method for the concentration of viruses from samples. When looking at studies in the literature that attempted to enrich for similar samples as ours, we determined that our strategy achieved the highest level of enrichment when estimating the proportion of viral sequences. Studies that employed centrifugation, filtration, and Nuclease treatments were below our level of enrichment [28,29]. In Paskey’s 2020 study, none of the aforementioned strategies were employed (or at least cited in the Materials and Methods), which could explain that this work reported the lowest level of enrichment. Interestingly, Salmier’s study of 2017 incorporated the use of a benchtop instrument for automated nucleic acid extraction, the NucliSENS easyMAG (Biomérieux). The instrument’s proprietary magnetic silica particle manipulation technology did not seem to impact viral nucleic acid enrichment, which is not surprising as it is not specifically targeted for this. Nucleic acid extraction methods utilized for these studies were similar except for the latter study. This suggests that this didn’t impact the level of enrichment. Our strategy included centrifugation, filtration, nuclease treatment, and in addition to this, negative enrichment through probe hybridization to select for host and microbial nucleic acids in an automated benchtop instrument. It is likely that Paskey also used a similar approach (albeit, without automation), as he used a previous version of the Illumina kit we used that likely includes probe negative enrichment. This is not specified in Table 1 as it is not explicit in the publication’s material and methods section. Despite this, it achieved lesser viral enrichment. The key to our approach resides in miniaturization of the reaction which improves kinetics paired with highly precise manipulation of magnetic beads, improving all cleanup steps. Our study shows that enrichment of such complex samples for virome studies can be improved utilizing a combination of traditional techniques including centrifugation, filtration, and nuclease treatment paired with molecular negative enrichment utilizing a mixture of probes. Automation, miniaturization, and precise bead handling thanks to a patented magnetic tweezer further improve enrichment and impact the resolution of the obtained data.

On the first level of resolution, the data generated in this study paints a detailed picture of the *M. chiloensis* virome. Unsurprisingly, most identified viruses are known to be associated with diverse insect species. This will ultimately enable us to gain insights into the diet of this bat species, which will in turn improve our knowledge on the role of *M. chiloensis* in the ecosystem and clarify its role as a pest-controlling mammal. Additionally, the resolution of the data obtained here could help indirectly survey insect population health, for example by identifying viruses affecting pollinator species. Although the dataset generated here is sufficient for this endeavor, this is not the main focus of this study and these analyses, although ongoing, are outside of the scope of this manuscript.

On a second level, the identification of diverse pathogenic viruses of zoonotic interest was made possible by the degree of enrichment of our study. Due to the fact that the sample tested positive for a pancoronavirus PCR test, this family of viruses was of particular interest. *De novo* assembly in parallel with taxonomic classification allowed for the identification of a novel alphacoronavirus sequence closely related to PEDV. A bat coronavirus sequence from *Tadarida brasiliensis* was linked with PEDV in Brazil [34], which reinforces the plausibility of our results.

In China, an alphacoronavirus, bat HKU2-like genetically distinct but clinically similar to PEDV was identified in diarrheic pigs and was designated swine acute diarrhea syndrome coronavirus (SADS-CoV) [35,36,37,38]; however, this genome sequence was phylogenetically far from alphacoronavirus detected in *M. chiloensis*. Furthermore, human alphacoronavirus 229E and Nl3 were not related to the alphacoronavirus genome identified in this study. This, however, should not minimize the relevance of determining its potential to jump to humans. Recently, a coronavirus isolated in Indonesian bats showed the capability of infecting human cells [39], highlighting once again the importance of sustaining this type of epidemiological surveillance. In addition to this, it should be noted that we did not detect sequences associated with any Rabies virus or coronavirus from the beta genera, underlining the absence of known zoonotic threats in this population. Our data improves the understanding of the viruses currently found in native chilean bats, highlighting the need to test the zoonotic potential of the virus identified through this study.

## Figures and Tables

**Table 1:** Percentage of viral sequences identified for this study compared to previous publications.

Publication	% Viral Sequences	Enrichment Method
Wu et al. [29]	0.8	Centrifugation, filtration, nuclease treatment, and QIAmp MinElute Virus Spin Kit
Salmier et al. [31]	0.3	Centrifugation, filtration, nuclease treatment, and NucliSENS easyMAG^®^ bio-robot
Wu et al. [28]	1.2	Centrifugation, filtration, nuclease treatment, and QIAamp viral RNA minikit
Paskey et al. [30]	0.226	QIAGEN RNeasy Kit with on-column DNase digestion
Aguilar et al., this study (2022)	2.2	Centrifugation, filtration, nuclease treatment, QIAamp viral RNA minikitand Magelia with Ribozero Plus

## Data Availability

The authors confirm the data supporting the findings of this study.

## Supplementary Materials

The following supporting information can be downloaded at: https://www.mdpi.com/article/10.3390/v14020202/s1, Table S1: Accession numbers for the viral sequences used for alignment/phylogenetic analyses in this study; Table S2: Significant identity values for generated contigs blasted in NCBI’s nr database; Table S3: All taxa identified through the Kaiju Web server tool; Supplementary file 1: IQTree full phylogeny result with SH-aLRT support (%)/ultrafast bootstrap support (%).
