# Supplementary material for: The Myotis chiloensis Guano Virome: Viral Nucleic Acid Enrichments for High-Resolution Virome Elucidation and Full Alphacoronavirus Genome Assembly
Source: Viruses. 2022 Jan 20;14(2):202. doi: 10.3390/v14020202 (PMC8875970; doi:10.3390/v14020202)
Supplement: Supplementary file 1 [file viruses-14-00202-s001.zip › Table S1.pdf]

Table S1: Genomes used for phylogenetic analyses in this study

Accession	Description	Size
NC_002645	Human coronavirus 229E, complete genome	27317
NC_005831.2	Human Coronavirus NL63, complete genome	27553
MK211369	Coronavirus BtSk-AlphaCoV/GX2018A, complete genome	28303
MK211370	Coronavirus BtSk-AlphaCoV/GX2018B, complete genome	28175
MK211371	Coronavirus BtSk-AlphaCoV/GX2018C, complete genome	28146
MK211372	Coronavirus BtSk-AlphaCoV/GX2018D, complete genome	28238
MK211373	Coronavirus BtRs-AlphaCoV/YN2018, complete genome	29109
NC_010437	Bat coronavirus 1A, complete genome	28326
NC_010438	Bat coronavirus HKU8, complete genome	28773
NC_009988	Bat coronavirus HKU2, complete genome	27165
KJ473806	BtMr-AlphaCoV/SAX2011, complete genome	27935
HQ728480	Cardioderma bat coronavirus/Kenya/KY43/2006	15474
NC_018871	Rousettus bat coronavirus HKU10, complete genome	28494
JQ989273	Hipposideros bat coronavirus HKU10 isolate TLC1347A, complete genome	28483
MF094687	Swine acute diarrhea syndrome related coronavirus isolate 141388, complete genome	27174

HQ728484	Miniopterus bat coronavirus/Kenya/KY27/2006	15314
MH938450	Alphacoronavirus Bat-CoV/P.kuhlii/Italy/206679-3/2010, complete genome	28146
MH938449	Alphacoronavirus Bat-CoV/P.kuhlii/Italy/3398-19/2015, complete genome	28128
MH938448	Alphacoronavirus Bat-CoV/P.kuhlii/Italy/206645-41/2011, complete genome	27862
KJ473795	BtMf-AlphaCoV/AH2011, complete genome	28310
KJ473796	BtMf-AlphaCoV/JX2012, complete genome	28311
KJ473797	BtMf-AlphaCoV/GD2012, complete genome	28758
KJ473798	BtMf-AlphaCoV/HuB2013, complete genome	28755
KJ473799	BtMf-AlphaCoV/FJ2012, complete genome	28765
KJ473800	BtMf-AlphaCoV/HeN2013, complete genome	28735
KJ473807	BtRf-AlphaCoV/HuB2013, complete genome	27608
KJ473808	BtRf-AlphaCoV/YN2012, complete genome	26975
KJ473809	BtNv-AlphaCoV/SC2013, complete genome	27783
KJ473810	BtMs-AlphaCoV/GS2013, complete genome	27576
HQ728486	Chaerephon bat coronavirus/Kenya/KY22/2006 polyprotein (ORF1ab)	15480
KT253270	229E-related bat coronavirus isolate BtCoV/FO1A-F2	28020
HQ728481	Chaerephon bat coronavirus/Kenya/KY41/2006	15578

NC_009020	Pipistrellus bat coronavirus HKU5	30482
NC_009021	Rousettus bat coronavirus HKU9	29114
NC_006577	Human coronavirus HKU1	29926
NC_001846	Murine hepatitis virus	31357
AY304488	Civet SARS CoV SZ16/2003	29731
NC_009694	Bat coronavirus HKU3	29728
DQ249224	Bat coronavirus HKU6	2780
DQ249228	Miniopterus bat coronavirus HKU8	2780
AF124991	Turkey coronavirus RNA-directed RNA polymerase (pol) gene	919
DQ648822	Bat coronavirus A515/2005	431
DQ648828	Bat coronavirus (BtCoV/A620/2005)	423
FJ710046	Bat coronavirus Hipposideros/GhanaKwam/19/2008	817
FJ710047	Bat coronavirus Hipposideros/GhanaKwam/20/2008	817
EU375854	Bat coronavirus M.das/Germany/D3.3/2007	405
EF544563	Rocky Mountain Bat Coronavirus 11	441

NC_009020	Pipistrellus bat coronavirus HKU5	30482
NC_009021	Rousettus bat coronavirus HKU9	29114
NC_006577	Human coronavirus HKU1	29926
NC_001846	Murine hepatitis virus	31357
AY304488	Civet SARS CoV SZ16/2003	29731
NC_009694	Bat coronavirus HKU3	29728
DQ249224	Bat coronavirus HKU6	2780
DQ249228	Miniopterus bat coronavirus HKU8	2780
AF124991	Turkey coronavirus RNA-directed RNA polymerase (pol) gene	919
